# Prediction of the Cause of Glaucoma Disease Identified by Glaucoma Optical Coherence Tomography Test in Relation to Diabetes and Hypertension at a National Hospital in Seoul: A Retrospective Study

**DOI:** 10.3390/diagnostics14131418

**Published:** 2024-07-03

**Authors:** Sun Jung Lee, Jae-Sik Jeon, Ji-Hyuk Kang, Jae Kyung Kim

**Affiliations:** 1 Department of Biomedical Laboratory Science, College of Health Sciences, Dankook University, Cheonan 31116, Republic of Korea; sunj0509@gmail.com (S.J.L.); zenty87@naver.com (J.-S.J.); 2 Department of Biomedical Laboratory Science, College of Health and Medical Science, Daejeon University, Daejeon 34520, Republic of Korea; shigella@naver.com

**Keywords:** glaucoma, optic nerve, retinal nerve fiber layer, ganglion cell–inner plexiform layer, optical coherence tomography, diabetes mellitus, hypertension

## Abstract

Glaucoma remains the primary cause of long-term blindness. While diabetes mellitus (DM) and hypertension (HTN) are known to influence glaucoma, other factors such as age and sex may be involved. In this retrospective study, we aimed to investigate the associations between age, sex, DM, HTN, and glaucoma risk. We employed optical coherence tomography (OCT) conducted using a 200 × 200-pixel optic cube (Cirrus HD OCT 6000, version 10.0; Carl Zeiss Meditec, Dublin, CA, USA). Effects obscured by low-test signals were disregarded. Data were amassed from 1337 patients. Among them, 218 and 402 patients had DM and HTN, respectively, with 133 (10%) exhibiting both. A sex-based comparison revealed slightly greater retinal nerve fiber layer (RNFL) and ganglion cell–inner plexiform layer (GCIPL) thickness in females. Patients without DM and HTN were predominantly in their 50 s and 60 s, whereas DM and HTN were most prevalent in those in their 60 s and 70 s. Both RNFL and GCIPL thicknesses decreased with advancing age in most patients. The study revealed that older individuals were more prone to glaucoma than younger individuals, with a higher incidence among patients with DM and HTN and reduced RNFL and GCIPL thicknesses. Furthermore, early detection before advancing age could furnish valuable preventive insights.

## 1. Introduction

Glaucoma is a complex condition characterized by structural alterations in the optic nerve. It is associated with functional impairments such as visual field defects, stemming from various risk factors, notably intraocular pressure (IOP) [1]. Moreover, the chronic and progressive nature of glaucomatous optic neuropathy results in significant damage to the optic nerve and the retinal nerve fiber layer (RNFL), culminating in permanent central or peripheral vision loss [2]. The identification of glaucomatous optic neuropathy hinges on discernible decreases in RNFL thickness around the optic disc. This is frequently accompanied by optic disc cupping, a phenomenon primarily attributed to ganglion cell and axonal changes. Early detection of RNFL defects, facilitated through the utilization of optical coherence tomography (OCT), is crucial. Alterations in RNFL thickness may precede overt visual impairment or the manifestation of visual field abnormalities in individuals with glaucoma [1].

In addition to assessing RNFL, OCT is a valuable tool for evaluating the ganglion cell–inner plexiform layer (GCIPL). In contrast to peripapillary RNFL measurements, previous research has underscored a stronger correlation between GCIPL thickness and the progression of structural glaucomatous damage, thereby enabling the prompt identification of axonal loss predicated on macular GCIPL measurements [3]. This highlights the critical role of OCT in the early detection and monitoring of glaucomatous pathology, providing insights that extend beyond conventional peripapillary RNFL assessments.

OCT is a groundbreaking non-invasive and non-contact imaging modality. Its resolution is unparalleled compared to other in vivo imaging techniques, such as scanning laser ophthalmoscopy, B-mode ultrasound, and ultrasound biomicroscopy, making it an indispensable tool for detecting retinal structure abnormalities [4]. Conceived by Huang et al. in 1991, OCT has undergone remarkable technological advancements over the years, resulting in enhancements in resolution and scanning speed, thereby further solidifying its position as a cornerstone in ocular imaging [1]. The utility of OCT extends far beyond mere imaging, as evidenced by its capability to accurately measure RNFL thickness with exceptional reproducibility and repeatability [1]. This reliability renders OCT an invaluable asset in the diagnosis, management, and longitudinal monitoring of various glaucoma and optic nerve disorders [1]. In clinical practice, OCT is increasingly employed as a tool for objective assessment and monitoring of RNFL, optic nerve head, and macular changes, facilitating precise evaluation of glaucoma progression [5]. The widespread adoption of OCT in ophthalmic practice underscores its versatility and utility in facilitating early diagnosis, guiding treatment decisions, and monitoring disease progression in patients with glaucoma and other optic nerve disorders. Through its high-resolution imaging capabilities and quantitative assessments, OCT continues to revolutionize the field of ophthalmology, empowering clinicians with invaluable insights into ocular pathology and facilitating more informed patient care.

Individuals with type 2 diabetes mellitus (DM) frequently present with various visual impairments, encompassing reduced color perception, diminished contrast sensitivity, slowed dark adaptation, decreased visual acuity, and compromised clarity of vision, despite the absence of overt clinical manifestations of diabetic retinopathy. These visual dysfunctions are intricately linked to the phenomenon of diabetic neurodegeneration [6]. Consequently, DM emerges as a potential contributor to the development of glaucomatous optic neuropathy, primarily through mechanisms involving elevated IOP, vasculopathy, or direct optic nerve damage [7].

Similarly, hypertension (HTN) is a significant risk factor for various ocular pathologies, elevating the propensity for conditions, including glaucoma, retinal vascular occlusion, retinal artery aneurysm, and ischemic optic neuropathy. Uncontrolled HTN can trigger the development of hypertensive retinopathy, characterized by manifestations such as macular edema, hard exudates, cotton wool spots, and retinal hemorrhage [6]. Notably, HTN, along with elevated IOP (even in individuals without a history of glaucoma), smoking, and a history of stroke, warrants consideration when evaluating RNFL thickness, as underscored by findings from a European study [8]. Moreover, a study conducted in Nepal reported a notable increase in the severity of glaucoma among individuals presenting with HTN, DM, or both, as evidenced by morphological and functional deficits [9].

These findings highlight the intricate interplay between systemic health conditions such as DM and HTN and the development and progression of glaucomatous optic neuropathy. By elucidating the multifactorial nature of glaucoma etiology, these insights underscore the importance of comprehensive ocular health assessments and the adoption of preventive measures aimed at mitigating the risk of vision-threatening complications associated with these systemic conditions.

The etiology of glaucoma encompasses a plethora of predisposing factors, many of which continue to elude complete understanding. Considering this complexity, this study aimed to uncover additional determinants that may exert influence on glaucoma beyond the established associations with DM and HTN. Specifically, we sought to elucidate the roles of age and sex in shaping the landscape of glaucomatous pathology. To achieve this objective, a comprehensive analysis was conducted using data collected from the ophthalmology department of a prominent national hospital in Korea. Through meticulous statistical examination of glaucoma OCT findings, focusing on measurements of RNFL and ganglion GCIPL in relation to DM and HTN status, we aimed to elucidate the multifaceted interplay between these variables and glaucoma susceptibility. By probing beyond the conventional risk factors, this study sought to broaden our understanding of the intricate mechanisms underlying glaucoma pathogenesis, paving the way for more targeted interventions and personalized management strategies.

## 2. Materials and Methods

### 2.1. Ethical Clearance

Ethical considerations serve as the cornerstone of any research endeavor, ensuring that investigations are conducted with integrity, respect for human dignity, and a commitment to upholding fundamental ethical principles. In accordance with these principles, the research design for the present study received approval from the Institutional Bioethics Committee of Dankook University (IRB approval number: 2022-08-010), thereby affirming its adherence to the guidelines set forth in the Declaration of Helsinki. It is noteworthy that patient consent was not deemed necessary for this study, as it utilized anonymized data obtained from diagnostic tests conducted within medical institutions. In accordance with established ethical guidelines and regulations governing research involving human subjects, the study’s methodology ensured the confidentiality and privacy of patients’ personal information. By leveraging anonymized data, the research team was able to analyze pertinent clinical information without compromising the privacy or autonomy of individual patients. This approach underscores the importance of safeguarding patient confidentiality while facilitating valuable insights into the subject matter under investigation.

In summary, the ethical clearance obtained from the Institutional Bioethics Committee of Dankook University, coupled with adherence to the principles of the Declaration of Helsinki, underscores the ethical rigor and integrity of the present study. The absence of patient consent, justified by the use of anonymized data, further reinforces the commitment to protecting patient confidentiality while advancing scientific knowledge in a responsible and ethical manner. The Institutional Bioethics Committee of Dankook University approved this study (IRB approval number: 2022-08-010), and it adhered to the principles of the Declaration of Helsinki. Patient consent was not required because the study used anonymized data from medical institutions’ diagnostic tests without accessing patients’ personal information.

### 2.2. Study Population

The study population comprised individuals seeking treatment for glaucoma at a prestigious national hospital located in Seoul, Republic of Korea, during the period spanning from 30 May 2022, to 24 June 2022. Additionally, individuals referred to the hospital specifically for glaucoma evaluation were also considered for inclusion in this retrospective study. Through a randomized selection process, a cohort of 1337 patients was identified and included in the analysis, with the aim of elucidating the relationship between glaucoma OCT test results and the presence of comorbidities such as diabetes mellitus (DM) and hypertension (HTN) (Table 1).

To ensure the reliability and validity of the data, results affected by low-test signal quality were meticulously excluded from the analysis. Subsequently, patients were categorized into three distinct groups based on their medical history and comorbidities: those presenting with both DM and HTN, those devoid of DM and HTN, and individuals falling into other categories. This stratification facilitated a comprehensive examination of the potential impact of these comorbid conditions on glaucoma OCT test outcomes.

In order to provide a nuanced understanding of the findings, statistical analyses were further stratified by age and sex. This approach allowed for the exploration of potential demographic variations in the relationship between glaucoma OCT test results and the presence of DM and HTN. Overall, the study population encompassed a diverse cohort of patients seeking glaucoma treatment or evaluation, with rigorous measures undertaken to ensure the integrity and validity of the data analyzed. Through comprehensive statistical analyses stratified by relevant demographic variables, the study sought to shed light on the complex interplay between glaucoma OCT test results and the presence of common comorbidities such as DM and HTN, thereby informing more targeted and effective approaches to glaucoma management and care.

### 2.3. OCT Examination Protocol

**Figure 1:**
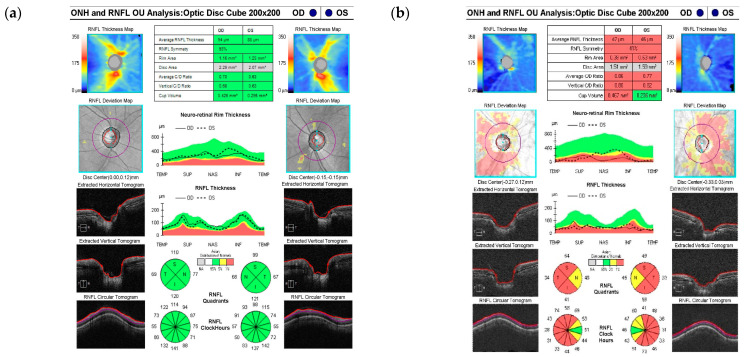
Result sheets of the RNFL test using optical coherence tomography showing (**a**) normal results and (**b**) glaucoma. The normal range is green, and the abnormal range is red. RNFL: retinal nerve fiber layer; ONH: optic nerve head; OU: oculus unitas, OD: oculus dexter; OS: oculus sinister; S: Superior; N: Nasal; I: Inferior; T: Temporal.

The OCT examination protocol utilized in this study involved the inclusion of patients who had undergone OCT imaging using the specified instrument. Specifically, optic nerve head scans were acquired utilizing a 200 × 200 optic cube on an OCT device (Cirrus HD OCT 6000, version 10.0; Carl Zeiss Meditec, Dublin, CA, USA). The optic disc cube scan was meticulously centered on the optic disc nerve head within the image, with an axial scan encompassing a 200 × 200-pixel frequency over a 6 × 6 mm area that spanned the optic nerve head and its adjacent surroundings (Figure 1).

**Figure 2:**
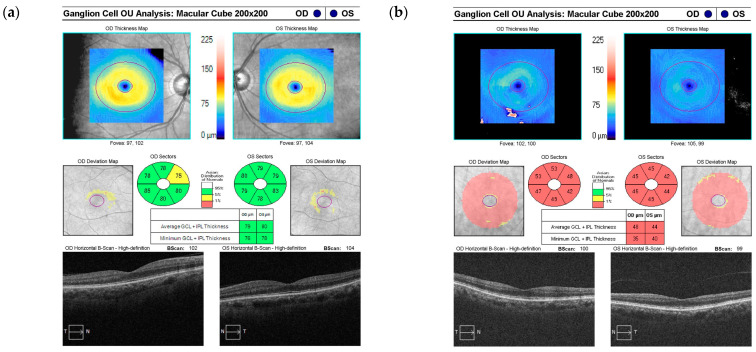
Result sheets of the GCIPL test using optical coherence tomography showing (**a**) normal results and (**b**) glaucoma. The normal range is green, and the abnormal range is red. GCIPL: ganglion cell–inner plexiform layer; OU: oculus unitas, OD: oculus dexter; OS: oculus sinister; S: Superior; N: Nasal; I: Inferior; T: Temporal.

Additionally, measurements of ganglion cell–inner plexiform layer (GCIPL) thickness were obtained through a macula cube scan, wherein the macula was positioned centrally within the image. Similar to the optic disc cube scan, the axial scan for the macula cube comprised a resolution of 200 × 200 pixels covering a 6 × 6 mm area, which encompassed the macula and its surrounding anatomical structures (Figure 2).

By adhering to this standardized OCT examination protocol, consistent and reliable imaging data were acquired for subsequent analysis. This meticulous approach ensured the comprehensive coverage of both the optic nerve head and the macula, facilitating a thorough assessment of relevant anatomical structures and parameters. Through the utilization of advanced imaging technology and standardized imaging protocols, the study aimed to obtain precise and clinically relevant OCT measurements, thereby contributing to a deeper understanding of the relationship between OCT findings and glaucoma pathophysiology.

### 2.4. Statistical Analysis

The statistical analysis conducted for this study involved a comprehensive approach encompassing data processing and analysis, with a focus on calculating average retinal nerve fiber layer (RNFL) and ganglion cell–inner plexiform layer (GCIPL) thicknesses. This analysis was facilitated through the utilization of MedCalc Software (MedCalc Software Version 20.218, Ostend, Belgium), a robust tool designed for statistical computation and analysis.

Prior to the commencement of statistical analysis, rigorous steps were taken to ensure the integrity and reliability of the data. This included the meticulous matching of participant characteristics such as age, sex, duration of diabetes mellitus (DM) and hypertension (HTN), and OCT findings. By aligning these key variables, the analysis aimed to minimize confounding factors and facilitate a more accurate interpretation of the results.

Once the data were appropriately curated and standardized, statistical analyses were performed using MedCalc Software. This involved the computation of average RNFL and GCIPL thickness measurements, allowing for a quantitative assessment of these key parameters across the study population. By quantifying these measurements, the analysis sought to elucidate any potential associations or correlations between RNFL/GCIPL thickness and the presence of DM and HTN.

Furthermore, the statistical analysis aimed to uncover any demographic or clinical factors that may influence the relationship between OCT findings and comorbidities such as DM and HTN. This comprehensive approach enabled a nuanced exploration of the data, shedding light on the complex interplay between these variables and their impact on glaucoma pathophysiology.

Overall, the statistical analysis conducted using MedCalc Software represented a critical component of the study, providing valuable insights into the relationship between OCT measurements and the presence of DM and HTN.

## 3. Results

During this study, comprehensive data analysis was conducted, encompassing records from a large cohort comprising 1337 patients, collected diligently between 30 May 2022 and 24 June 2022. Within this cohort, 218 individuals were identified as having DM, 402 individuals were diagnosed with HTN, and a notable subset of 133 patients (constituting 10% of the total cohort) presented with both conditions concurrently. Subsequent grouping of patients based on their DM and HTN status—stratified into categories including those devoid of DM and HTN, those affected by both DM and HTN, and the entirety of patients amalgamated—yielded findings showcasing minimal discrepancies in RNFL and ganglion GCIPL thicknesses.

However, the comparative analysis based on sex showed that the RNFL and GCIPL were slightly thicker in female patients compared to male patients but without a statistically significant difference. Among them, the thinnest RNFL and GCIPL thicknesses were in a male patient with HTN (Table 2). Moreover, meticulous scrutiny of the results derived from the RNFL and GCIPL tests utilizing OCT revealed noticeable differences between patients without glaucoma (group A) and those diagnosed with glaucoma (group B) (Figure 1 and Figure 2).

Regarding age distribution within the studied population, a notable trend emerged, delineating distinct patterns among patients categorized based on their DM and HTN status. Specifically, individuals without both DM and HTN were predominantly clustered within the age range spanning from their 50 s to 60 s. Conversely, among patients with both DM and HTN concurrently, a notable shift was observed in age distribution, with a predominant representation observed among individuals in their 60 s and 70 s (Table 3).

**Figure 3:**
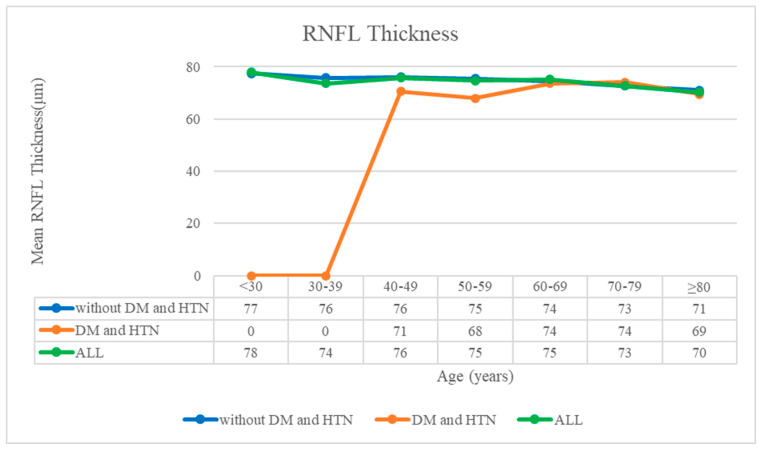
Correlation between the mean retinal nerve fiber layer thickness and age. The blue, orange, and green lines indicate patients without DM and HTN, patients with DM and HTN, and all patients (including individuals with only DM or only HTN), respectively. DM: diabetes mellitus; HTN: hypertension; RNFL: retinal nerve fiber layer.

The analysis of RNFL thickness in relation to age yielded compelling insights, revealing a consistent trend of decreased RNFL thickness with advancing age across all patient subgroups under study. Whether considering patients without both DM and HTN, those with DM and HTN alone, or the entire patient cohort collectively, RNFL thickness significantly decreased (Figure 3). This consistent pattern underscores the pervasive impact of aging on eye health and function.

**Figure 4:**
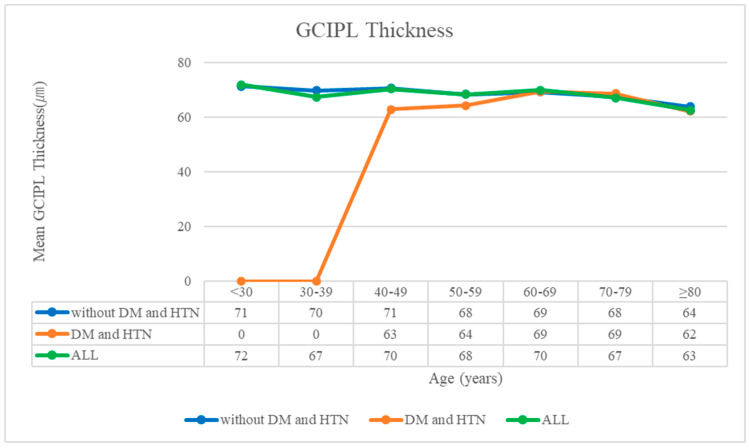
Correlation between the mean ganglion cell–inner plexiform layer thickness and age. The blue, orange, and green lines indicate patients without DM and HTN, patients with DM and HTN, and all patients (including individuals with only DM or only HTN), respectively. DM: diabetes mellitus; GCIPL: ganglion cell–inner plexiform layer; HTN: hypertension.

Similarly, the examination of ganglion GCIPL thickness revealed a parallel trend, with diminishing GCIPL thickness observed across all patient subgroups delineated by DM and HTN status, as well as across the entire patient cohort. Irrespective of the presence or absence of DM and HTN, the impact of advancing age on GCIPL thickness remained unequivocal (Figure 4). These findings underscore the multifaceted effects of aging on retinal structures beyond just the RNFL, underscoring the importance of age as a fundamental determinant in the progression of ocular pathology. By elucidating the age-related changes in both RNFL and GCIPL thickness, this study contributes valuable insights into the physiological aging processes affecting retinal health and function, thereby informing more targeted strategies for the early detection and management of age-related ocular conditions, such as glaucoma.

## 4. Discussion

Glaucoma, a leading cause of irreversible blindness, can manifest at any age and is marked by progressive optic neuropathy, which entails the loss of retinal ganglion cells and thinning of the RNFL [10,11]. Assessing peripapillary RNFL thickness aids in detecting glaucomatous structural changes and early diagnosis of glaucoma, while GCIPL thickness provides additional diagnostic accuracy [11,12]. In this study, we explored RNFL and GCIPL thickness in relation to age, sex, and their association with DM and HTN to assess their impact on glaucoma development. While DM and HTN exhibited age-related trends, RNFL and GCIPL thickness were primarily influenced by age rather than by DM and HTN, with subtle sex-related differences observed.

After stratifying based on DM and HTN status, our analysis revealed minimal disparities in RNFL and ganglion GCIPL thickness among patients with varying medical conditions. However, a nuanced examination of age distribution within the studied population unveiled intriguing patterns as follows: patients without both DM and HTN were predominantly clustered within the age range spanning from their 50 s to 60 s, whereas those with both DM and HTN concurrently exhibited a notable prevalence among individuals in their 60 s and 70 s. These age-related trends underscore the complex interplay between systemic health conditions and aging processes, with implications for disease susceptibility and progression. Notably, insights gleaned from the International Diabetes Federation Atlas Guidelines Report emphasize the heightened risk of developing DM among individuals with impaired glucose tolerance, with projections suggesting a concerning increase in DM prevalence by 2045 [13]. Furthermore, previous research focusing on hypertensive disorders during pregnancy has highlighted the significance of addressing HTN as a health concern spanning both the perinatal period and later stages of life [14]. Additionally, prior studies have underscored the strong association between older age and the incidence of both DM and HTN, further highlighting the intricate relationship between age and systemic health conditions [15].

These findings underscore the multifaceted nature of disease epidemiology, highlighting the importance of considering demographic variables and systemic health status in understanding disease patterns and guiding preventive interventions. By elucidating these intricate relationships, our study contributes to a deeper understanding of the complex interplay among age, systemic health conditions, and ocular health outcomes, thereby informing more targeted approaches to disease prevention and management.

The meticulous statistical analysis conducted in this study unearthed compelling trends regarding RNFL and ganglion GCIPL thickness in relation to age. Consistent with findings from prior research in Germany and Taiwan [16,17], our analysis revealed a significant decline in RNFL thickness with advancing age. This age-related thinning of the RNFL was further underscored by observations of older adults with HTN and DM, who exhibited notably thinner average RNFL measurements [18]. These findings highlight the profound influence of age on retinal health and structure, with implications for the pathophysiology of ocular conditions such as glaucoma. Similarly, our analysis revealed a consistent decrease in GCIPL thickness with age, aligning with evidence from previous studies documenting age-related thinning of the ganglion cell complex [19,20,21]. This age-related decline in GCIPL thickness further emphasizes the multifaceted effects of aging on retinal morphology and function, with potential implications for visual health and disease susceptibility. By corroborating these findings with those in the existing literature, our study contributes to a deeper understanding of the complex interplay between age and retinal structure, paving the way for more targeted approaches to disease prevention and management in older populations.

In our comprehensive study, using data sourced from national hospitals in Korea, we conducted a detailed analysis of RNFL, ganglion GCIPL, and OCT test results in conjunction with DM and HTN data, meticulously stratified based on age and sex. The rich dataset enabled a nuanced exploration of the intricate relationships among demographic variables, systemic health conditions, and ocular health outcomes. The findings gleaned from our analysis revealed noteworthy associations between older age and a heightened prevalence of both DM and HTN, coinciding with diminished RNFL and GCIPL thickness. This convergence of factors underscores the augmented risk of glaucoma development with advancing age, highlighting the imperative of early diagnosis and effective management strategies before the onset of age-related ocular pathologies [22]. Early detection facilitated by non-invasive OCT examination emerges as a crucial component in this endeavor, offering reliable early indicators of glaucomatous risks through assessments of RNFL and GCIPL thickness. Using these predictive metrics, clinicians can proactively identify individuals at heightened risk of visual field defects, thereby enabling timely intervention and preservation of visual function [23].

These findings underscore the significance of preventive management strategies, advocating for regular glaucoma examinations encompassing assessments of RNFL and GCIPL thickness. By implementing proactive measures aimed at early detection and intervention, clinicians can effectively mitigate the progression of glaucoma and minimize the associated risk of irreversible vision loss. Through such concerted efforts, we can strive toward optimizing visual health outcomes and enhancing the quality of life for individuals at risk of glaucoma and related ocular conditions.

Our study, conducted using data from national hospitals in Korea, analyzed RNFL, GCIPL, and OCT test results alongside DM and HTN data, stratified based on age and sex. The findings indicated that older age was associated with a higher prevalence of DM and HTN, as well as lower RNFL and GCIPL thickness, indicating an increased risk of glaucoma with age. Early diagnosis and effective treatment before advancing age can delay vision loss owing to glaucoma [22]. Non-invasive OCT examination provides reliable early indicators of glaucomatous risks, such as RNFL and GCIPL thickness, aiding in predicting subsequent visual field defects [23]. These findings underscore the importance of preventive management through regular glaucoma examinations for RNFL and GCIPL thickness.

This study had some limitations, including its retrospective nature, precluding consideration of patient clinical characteristics such as obesity and genetic diseases. Second, sufficient sample values must be obtained from each age group, especially young age groups. Lastly, the study was conducted within a short timeframe and limited to one region in Korea, potentially affecting the generalizability of the findings. Therefore, further extensive studies in diverse regions are warranted.

## 5. Conclusions

The insights gleaned from our study underscore the complex interplay between diabetes mellitus (DM), hypertension (HTN), age, and sex in shaping the risk landscape for glaucoma development. While our findings elucidate the potential contributions of DM and HTN to the pathogenesis of glaucomatous optic neuropathy, they also underscore the pivotal role of age as a predominant risk factor for glaucoma susceptibility. Despite the presence of subtle sex-related differences, age emerges as a critical determinant in the progression and severity of glaucoma, highlighting the imperative of age-specific interventions and tailored management strategies.

This study underscores the importance of comprehensive assessment and regular monitoring of RNFL and GCIPL thicknesses, particularly in older populations and those with concurrent DM and HTN, to facilitate timely intervention and prevent irreversible vision loss.

## Figures and Tables

**Table 1:** Number of patients categorized by age and sex.

Age Group (Years)	Male	Female	Total
<30	16	21	37
30–39	27	22	49
40–49	70	91	161
50–59	126	102	228
60–69	178	210	388
70–79	138	192	330
≥80	65	79	144

**Table 2:** Differences in RNFL and GCIPL thickness according to the status of the patients and sex.

	Sex	RNFL Thickness (µm)	GCIPL Thickness (µm)
Patients without DM and HTN	Male	73	68
	Female	76	69
Patients with DM and HTN	Male	72	67
	Female	73	67
DM	Male	74	69
	Female	76	70
HTN	Male	69	65
	Female	75	67
All patients	Male	72	68
	Female	76	69

DM: diabetes mellitus, HTN: hypertension, RNFL: retinal nerve fiber layer, GCIPL: ganglion cell–inner plexiform layer.

**Table 3:** Number of patients with or without DM and HTN by age (years).

Age Group (Years)	<30	30–39	40–49	50–59	60–69	70–79	≥80
Without DM and HTN	35	43	135	175	246	159	57
DM	0	0	14	21	64	81	38
HTN	2	6	20	42	115	142	75
DM and HTN	0	0	8	10	37	52	26

DM: diabetes mellitus; HTN: hypertension. The total for each group is not the total number of people in that group.

## Data Availability

The raw data supporting the conclusions of this article will be made available by the authors upon request.
